# C/D box small nucleolar RNA SNORD104 promotes endometrial cancer by regulating the 2ʹ-O-methylation of *PARP1*

**DOI:** 10.1186/s12967-022-03802-z

**Published:** 2022-12-24

**Authors:** Bingfeng Lu, Xi Chen, Xin Liu, Jingwen Chen, Honglei Qin, Shuo Chen, Yang Zhao

**Affiliations:** Department of Obstetrics and Gynecology, Department of Gynecologic Oncology Research Office, Guangdong Provincial Key Laboratory for Major Obstetric Diseases, The Third Affiliated Hospital of Guangzhou Medical University, No. 63 Duobao Raod, Liwan District, Guangzhou, 510150 Guangdong People’s Republic of China

**Keywords:** SNORD104, 2ʹ-O-methylation, Fibrillarin, *PARP1*, Endometrial cancer

## Abstract

**Background:**

Small nucleolar RNAs (snoRNAs) are dysregulated in many cancers, although their exact role in tumor genesis and progression remains unclear.

**Methods:**

The expression profiles of snoRNAs in endometrial cancer (EC) tissues were analyzed using data from The Cancer Genome Atlas, and SNORD104 was identified as an upregulated snoRNA in EC. The tumorigenic role of SNORD104 in EC was established in CCK8, colony formation, EdU, apoptosis, Transwell, and in vivo xenograft experiments. The molecular mechanisms of SNORD104 were analyzed by RNA immunoprecipitation (RIP), Nm-seq, RTL-P assay, RNA stability assay, qRT-PCR, and western blotting.

**Results:**

Antisense oligonucleotide (ASO)-mediated knockdown of SNORD104 in Ishikawa cells significantly inhibited their proliferation, colony formation ability, migration, and invasion in vitro and increased apoptosis. On the other hand, overexpression of SNORD104 promoted EC growth in vivo and in vitro. RIP assay showed that SNORD104 binds to the 2ʹ-O-methyltransferase fibrillarin (FBL), and according to the results of Nm-seq and RTL-P assay, SNORD104 upregulated *PARP1* (encoding poly (ADP-ribose) polymerase 1) 2ʹ-O-methylation. The binding of FBL to *PARP1* mRNA was also verified by RIP assay. Furthermore, SNORD104 expression was positively correlated with *PARP1* expression in EC tissues. In the presence of actinomycin D, SNORD104 increased the stability of *PARP1* mRNA and promoted its nuclear localization. Finally, silencing *FBL* or *PARP1* in the HEC1B cells overexpressing SNORD104 inhibited their proliferative and clonal capacities and increased apoptosis rates.

**Conclusions:**

SNORD104 enhances *PARP1* mRNA stability and translation in the EC cells by upregulating 2ʹ-O-methylation and promotes tumor growth.

**Supplementary Information:**

The online version contains supplementary material available at 10.1186/s12967-022-03802-z.

## Introduction

Endometrial cancer (EC) is one of the most common malignancies in menopausal and postmenopausal women in developed countries, and its incidence is increasing worldwide [1, 2]. Although surgery, chemotherapy, and radiotherapy have achieved satisfactory outcomes, the survival rate of EC patients is still dismal due to the tendency of the tumor cells to relapse and metastasize [3]. Therefore, it is crucial to elucidate the molecular mechanisms underlying the initiation, progression, and metastasis of EC in order to identify potential therapeutic targets for personalized treatment. Small nucleolar RNAs (snoRNAs) are a class of small non-coding RNAs (ncRNAs) with conserved structural elements that are widely distributed in the nucleolus of eukaryotic cells and broadly classified into the C/D box snoRNAs (SNORDs) and H/ACA box snoRNAs [4]. They are usually encoded by intron regions of coding genes or non-coding genes and are complicated by the transcription and processing of host genes, with only a few being encoded by independent genomic locations [5]. The snoRNAs were long considered to be involved in the nucleolar function. Recent studies show that snoRNAs mediate 2ʹ-O-methylation and pseudouridylation of rRNA, tRNA, mRNA, snRNA, and other RNAs via complementary base pairing and also regulate gene expression by forming snoRNP complexes with ribonucleolar proteins (RNPs) [6, 7]. We identified the snoRNAs potentially related to the development of EC by screening the transcriptomic data of The Cancer Genome Atlas (TCGA) database. SNORD104 (located at 17q23.3, also known as U104) was significantly upregulated in the EC tissues, although its role in cancer, particularly EC, remains unclear.

Fibrillarin (FBL) is a component of the small nucleolar ribonucleoprotein (snRNP) (including FBL, NOP56, NOP58) that contains N-terminal repeat domains rich in glycine and arginine residues, similar to the FBLs in other species. The central region of FBL resembles an RNA binding domain and contains a common RNP sequence [8]. FBL is a key 2ʹ-O-ribose methylase that catalyzes SNORD-dependent methylation of target RNAs [9]. However, whether FBL-catalyzed 2ʹ-O-methylation plays an important role in EC remains unknown. In this study, we evaluated the oncogenic effects of SNORD104 in EC through established in vitro and in vivo experiments, and explored the molecular mechanisms by 2ʹ-O-methylation sequencing (Nm-seq) and RNA immunoprecipitation.

## Methods

### Human tissue samples

Tumor tissue samples were collected from 71 treatment-naïve EC patients who underwent surgery at the Third Affiliated Hospital of Guangzhou Medical University. The samples were snap frozen in liquid nitrogen immediately after resection and stored at − 80 °C. The study protocol was approved by the ethics committee of the hospital, and all patients provided written informed consent.

### Cell culture and transfection

Human EC cell lines (Ishikawa, HEC1A, HEC1B, and KLE) and immortalized endometrial cells (EEC) were purchased from ATCC (Manassas, VA, USA) or Jennio Biotech (Guangzhou, China). HEC1B cells were cultured in DMEM (HyClone, Logan, UT, USA), HEC1A and Ishikawa cells in RPMI-1640 medium (HyClone), and the KLE cells in DMEM-F12 (HyClone). All media were supplemented with 10% fetal bovine serum (FBS; Gibco, Grand Island, NY, USA) and 100 U/ml penicillin and streptomycin (Gibco). The cell lines were incubated at 37 °C under 5% CO_2_. As per the experimental requirements, the cells were transfected with SNORD104 ASO (TCTTTCTCGTAAATGCTGAG), si-*FBL* (GGGCTAAGGTTCTCTACCT), or si-*PARP1* (CACGGACTCTCTACCGTAT) using Lipofectamine 3000 (Invitrogen, Carlsbad, CA, USA) according to the manufacturer’s instructions. The oligonucleotides were synthesized by RIBOBIO (Guangzhou, China).

### Bioinformatics analysis

The clinical and transcriptomic data of EC were downloaded from https://portal.gdc.cancer.gov/projects/TCGA-UCEC, ITPR3, LMNB1, PARP1, PARP4, and TUBA1C protein expression in EC tissues was analyzed using the CPTAC [10] database (http://ualcan.path.uab.edu). The correlation between PARP1 expression and prognosis of EC patients was evaluated by Kaplan–Meier survival analysis using KMplot [11] (http://kmplot.com).

### Cell viability assays

The cells were seeded in a 96-well plate at the density of 2000 cells/100 μl per well and transfected with the respective constructs. The CCK8 reagent (Yeasen Biotechnology, Shanghai, China) was added to the cells at 0, 24, 48, and 72 h, and the absorbance of each well was measured at 450 nm using a microplate reader (BioTek, Winooski, VT, USA). The experiment was repeated thrice.

### Colony formation assay

The cells were seeded in a 6-well plate at the density of 500 cells/2 ml per well and transfected with the respective constructs once they adhered. After culturing for 7–10 days, the colonies were fixed using formaldehyde for 15 min, washed thrice with PBS, and stained with crystal violet for 10 min. The plates were rinsed with tap water to remove the excess dye and dried, and the number of colonies was counted.

### EdU assay

The Ishikawa/HEC1B cells were seeded in 96-well plates, and EdU solution (Thermo Scientific, USA) was added. The cells were incubated for 16 h, and the subsequent steps were followed as per the manufacturer's instructions. The stained cells were observed under a microscope (Olympus, Tokyo, Japan).

### Apoptosis assay

Apoptosis was detected by propidium iodide (PI) and fluorescein isothiocyanate (FITC)-Annexin V (BD Pharmingen, San Diego, CA, USA) staining according to the manufacturer's instructions. Briefly, the cells were harvested 48 h after transfection and washed twice with cold PBS. The suspension was stained with 100 μl 1 × buffer and 5 μl FITC-Annexin V and PI in the dark for 15 min. The reaction was terminated by adding 400 μl 1 × buffer, and the cells were analyzed by flow cytometry within 1 h.

### Quantitative real‐time reverse transcription PCR

Total RNA was extracted from EC cell lines or tissues using TRIzol reagent (1 ml; Takara, Shiga, Japan) and reverse transcribed to cDNA. Quantitative real-time PCR was performed using the SYBR PreMix Ex TAQ II kit (Takara). The primer sequences were as follows: U6: forward: 5ʹ CTCGCTTCGGCAGCACA 3ʹ; reverse: 5ʹ AACGCTTCACGAATTTGCGT 3ʹ; *GAPDH*: forward: 5ʹ CCCATCACCATCTTCCAGGAG 3ʹ; reverse: 5ʹ GTTGTCATGGATGACCTTGGC 3ʹ; SNORD104: forward: 5ʹ CATTCCAATTAAAGCACG 3ʹ; reverse: 5ʹ CAGACTCCAGTTCGCATC 3ʹ; *PARP1*: forward: 5ʹ CGGAACAAGGATGAAGTGAA 3ʹ; reverse: 5ʹ TTGGTGGAGGCGGAGA 3ʹ; *PARP4*: forward: 5ʹ TAGCCTGGTCATTTGGT 3ʹ; reverse: 5ʹ AGATGGCATTCTTCACG 3ʹ; 18S: forward: 5ʹ GAAACGGCTACCACATCC 3ʹ; reverse: 5ʹ ACCAGACTTGCCCTCCA 3ʹ; *ITPR3*: forward: 5ʹ CCAAGCAGACTAAGCAGGACA 3ʹ; reverse: 5ʹ ACACTGCCATACTTCACGACA 3ʹ; *LMNB1*: forward: 5ʹ AAGCATGAAACGCGCTTGG 3ʹ; reverse: 5ʹ AGTTTGGCATGGTAAGTCTGC 3ʹ; *TUBA1C*: forward: 5ʹ TGTTTGTAGACTTGGAACCCAC 3ʹ; reverse: 5ʹ GCCAATGGTGTAGTGCCCT 3ʹ.

### Western blotting

Total protein was extracted from cultured cells or tissues using radioimmunoprecipitation assay (RIPA) buffer containing protease inhibitors and quantified using the BCA Protein Assay Kit (Beyotime, China). The protein samples were diluted with the appropriate amount of loading buffer and PBS to 2 μg/l, and denatured by heating at 95 °C for 15 min. The samples were resolved by 10% or 12% sodium dodecyl sulfate polyacrylamide gel electrophoresis (SDS-PAGE) and transferred to polyvinylidene fluoride (PVDF) membranes (Amersham, Munich, Germany). Following overnight incubation with rabbit anti-FBL (1:2000 Magna, Proteintech, Rosemont, IL, USA), rabbit anti-PARP1 (1:2000 Magneto, Proteintech), rabbit anti-PARP4 (1:2000, Bioss, Woburn, MA, USA), anti-ITPR3 (1:2000, LSBio), anti-LMNB1 (1:10,000, Proteintech), anti-Vinculin (1:10,000, Proteintech), anti-GAPDH (1:5000, Proteintech) and anti-α-tubulin (TUBA1B/1C) (1:2000, Proteintech) antibodies at 4 ℃, the membranes were washed thrice with 1% Tris-buffered saline-Tween20 (TBST) buffer for 10 min each time. The membranes were then incubated with the secondary antibody for 2 h, and washed again with 1% TBST buffer for 10 min. The bands were visualized using an enhanced chemiluminescence system (NCM Biotech, Suzhou, China), and the protein levels were quantified using grayscale values.

### RNA immunoprecipitation (RIP) assay

RIP assay was performed using the Magna RIP RNA-Binding Protein Immunoprecipitation Kit (Millipore, Bedford, MA, USA) as per the manufacturer's instructions. Cells growing at 80–90% confluence were harvested and lysed using RIPA lysis buffer. The cell extracts were incubated with magnetic beads with anti-mouse FBL (Santa Cruz Biotechnology, Santa Cruz, CA, USA) or normal mouse IgG (negative control) in the RIPA lysis buffer. After digesting the protein using protease K, the immunoprecipitated RNA was isolated and analyzed using qRT-PCR.

### Nm-seq analysis

Nm-sequencing was performed by the Shanghai Cloud-seq Biotech Co. Ltd. as previously described [12] using the Illumina NovaSeq 6000 sequencer.

### Reverse transcription at low dNTPs-PCR (RTL-P) assay

RTL-P assay was performed to detect the 2′-O-methylation level (Nm) of PARP1 according to published methods [13, 14]. Briefly, 5 μg total RNA was incubated with low (1 μM) or high (1 mM) concentration of dNTPs (Takara) and 1 μl specific RT primers at 65 °C for 5 min and then placed on ice. The reaction was performed using 4 μl M-MLV RT 5 × buffer (PROMEGA), 1 μl 200 U/μl M-MLV Reverse Transcriptase (PROMEGA), 1 μl 0.1 M DTT (PROMEGA), and 1 μl 40 U/μl RNase inhibitor (PROMEGA) at 50 °C for 1 h, and then at 70 °C for 15 min. QRT-PCR was performed on the SYBR Prime X Ex-TAQ Patent II Suite (Takara) using PARP1-specific primers. The relative expression of the gene was calculated by comparing the period threshold (Ct) of the target gene in the experimental group and the control group at low concentrations according to the 2^−ΔCt^ method.

### RNA stability assay

Stably transfected cells were seeded into a 6-well plate and incubated until they reached 70% confluence. Actinomycin D (Sigma, St. Louis, MO, USA) was added (Act D, 5 µg/ml), and the cells were harvested at 0, 6, and 12 h. Total RNA was extracted using TRIzol reagent, and *PARP1* mRNA levels were detected by qRT-PCR.

### Nucleocytoplasmic separation assay

The confluent cells were harvested using a scraper and centrifuged at 1500 rpm for 5 min. The supernatant was removed, and the nuclear and cytoplasmic fractions were extracted from the cells using a specific kit (Beyotime, Shanghai, China). Both fractions were analyzed by western blotting as per standard protocols.

### Nude mouse xenograft assay

All animal experiments were conducted in accordance with the guidelines of the National Institute of Health on the Care and Use of Experimental Animals and were approved by the Animal Care and Use Committee of Guangzhou Medical University. Four-week-old female BALB/c nude mice were purchased from Guangdong Experimental Animal Center (Foshan, China) and housed under controlled temperature and light (12 h dark/12 h light) with ad libitum access to food and water. The mice were injected subcutaneously into their right flanks with 1 × 10^7^ tumor cells suspended in 200 μl of FBS-supplemented culture medium. The tumors were measured twice a week, and the volume was calculated according to the formula (length × width^2^)/2. At the end of the experiment (or when the mice died), the mice were euthanized, and the tumors were excised.

### Statistical analysis

GraphPad Prism [version 8.02(263)] was used for statistical analysis. Non-parametric Mann–Whitney test for small samples that do not meet the normal distribution; Student's t-test was used for the normal distribution and when the sample size was large; Correlations between groups were determined using the Pearson correlation coefficient. The CCK-8 assay data were analyzed using ANOVA. All data were presented as the mean ± S.D of at least three independent experiments. Statistical significance was set at P < 0.05.

## Results

### SNORD104 is upregulated in EC tissues

**Fig. 1:**
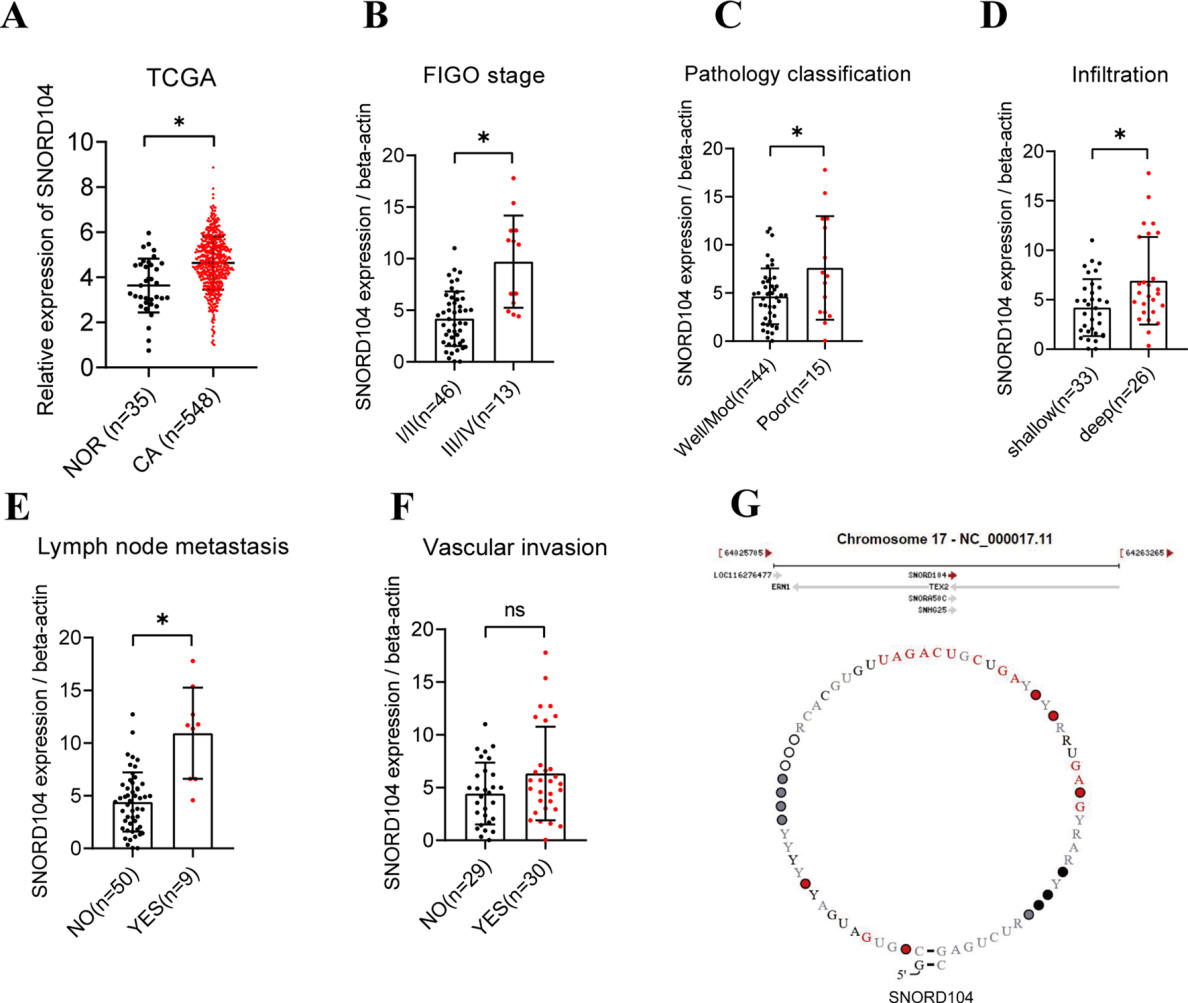
SNORD104 is upregulated in EC tissues and associated with clinicopathological features. **A** TCGA data set analysis showing SNORD104 expression levels in EC tissue samples (n = 548) and normal endometrial tissue samples (n = 35). **B**–**F** Correlation of SNORD104 with FIGO stage, differentiation, myometrial invasion, lymph node metastasis, and vascular invasion. **G** Schematic and genomic location of SNORD104. Data were calculated and shown as the mean ± SD (error bars) from three independent experiments. *P < 0.05, ns means P > 0.05

We analyzed the transcriptomic profiles of 548 EC tissue samples and 35 normal endometrial tissue samples from TCGA datasets (Fig. 1A), and found that SNORD104 was upregulated in the EC samples. To verify these results, we analyzed the expression of SNORD104 in post-operative EC tissues collected from the Third Affiliated Hospital of Guangzhou Medical University and analyzed the correlation between the expression of SNORD104 and clinicopathological features (Additional file 1: Table S1). SNORD104 expression was higher in patients with stage III/IV disease (n = 13) compared to those with stage I/II disease (n = 46) (Fig. 1B). In addition, SNORD104 was upregulated in the poorly differentiated (n = 15) versus the moderately or well-differentiated (n = 44) tumors (Fig. 1C). SNORD104 expression was also higher in patients with deep myometrial invasion (n = 26) compared to those with shallow myometrial invasion (n = 33) (Fig. 1D), as well as in patients with lymph node metastasis (n = 26) relative to those without lymph node metastasis (n = 33) (Fig. 1E). In contrast, there was no significant correlation between SNORD104 expression and vascular invasion (Fig. 1F). Therefore, we hypothesized that SNORD104 functions as an oncogene in EC.

### *SNORD104 knockdown inhibited the malignant potential of EC cells *in vitro

**Fig. 2:**
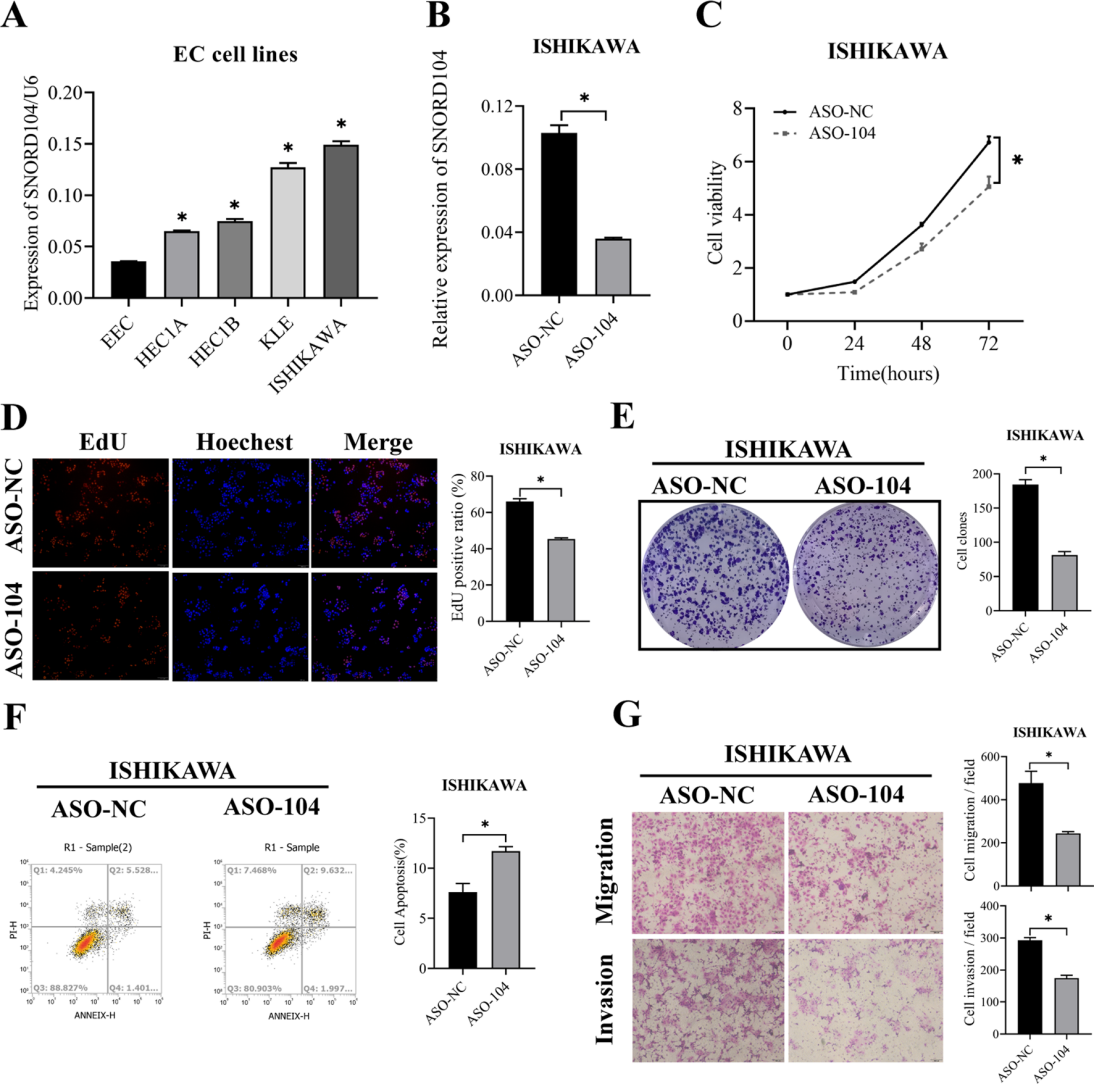
SNORD104 knockdown repressed EC growth in vitro. **A** SNORD104 expression levels in EC cell lines and immortalized endometrial EEC cells. **B** Transfection of ASO in Ishikawa cells inhibited SNORD104 levels. **C** Viability, **D** Proliferation rates, **E** Colony formation, **F** apoptosis rates, and **G** migration and invasion of EC cells with SNORD104 knockdown. Data were calculated and shown as the mean ± SD (error bars) from three independent experiments. *P < 0.05

Genome sequence analysis showed that SNORD104 was processed from the first intron region of the primary transcript of the *SNHG25* gene (small nucleolar RNA host gene 25, located at 17q21.3) (Fig. 1G), and the mature *SNHG25* RNA was transcribed from the exons. We found that *SNHG25* was also upregulated in EC tissues (Additional file 2: Fig. S1A, *P < 0.05). In addition, SNORD104 was upregulated in the EC cell lines compared to that in the immortalized endometrial cells. Both SNORD104 and *SNHG25* were highly expressed in the Ishikawa cells, but showed relatively low expression levels in the HEC1B and HEC1A cells (Fig. 2A, Additional file 2: Fig. S1B, *P < 0.05). Previous studies have shown that the host genes of snoRNAs regulate their respective snoRNAs and play a role in tumor development [15, 16]. To determine the role of SNORD104 and *SNHG25* in EC cells, we respectively transfected the Ishikawa cells with *SNHG25*-specific small interfering RNA (siRNA) and the HEC1B cells with SNHG25-overexpression plasmid, and an antisense oligonucleotide sequence (ASO) targeting SNORD104 in HEC1B cells. The knockdown efficiency was verified by qRT-PCR (Fig. 2B, Additional file 2: Fig. S1C, D, *P < 0.05). SNORD104 knockdown significantly decreased the viability, proliferation (Fig. 2C, D, *P < 0.05), clonal capacity (Fig. 2E, P < 0.05), migration, and invasion (Fig. 2G, *P < 0.05) of Ishikawa cells, and increased the apoptosis rates (Fig. 2F, *P < 0.05). In contrast, knocking down or overexpressing *SNHG25* had almost no effect on the proliferation and apoptosis of endometrial cancer cells (Additional file 2: Fig. S1E–H, ns means P > 0.05).

### *SNORD104 promotes EC progression *in vitro* and *in vivo

**Fig. 3:**
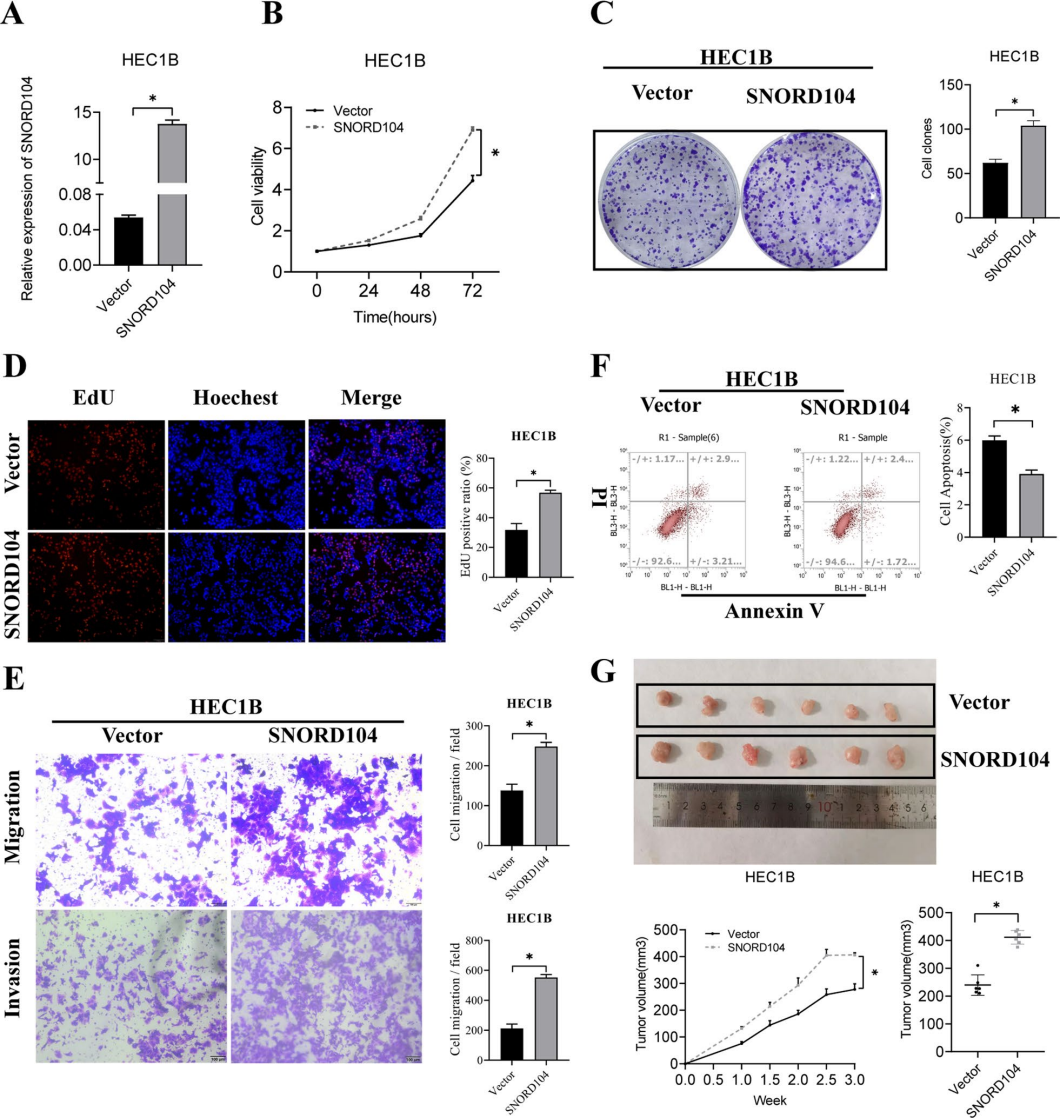
SNORD104 overexpression promoted EC growth in vitro and in vivo. **A** SNORD104 expression in HEC1B cells transfected with the overexpression plasmid. **B** Viability, **C** colony formation, **D** proliferation rates, **E** apoptosis rates, and **F** migration and invasion of HECB1 cells overexpressing SNORD104. **G** Tumor volumes in nude mice inoculated with control or SNORD104-overexpressing HEC1B cells. Data were calculated and shown as the mean ± SD (error bars) from three independent experiments. *P < 0.05

HEC1B cells stably transfected with the SNORD104 plasmid (Fig. 3A, *P < 0.05) exhibited high proliferative capacity (Fig. 3B–D, *P < 0.05), along with increased invasion and migration rates (Fig. 3E, *P < 0.05). Furthermore, SNORD104 overexpression reduced apoptosis rates of HEC1B cells (Fig. 3F, *P < 0.05). Consistent with the in vitro results, HEC1B cells stably expressing SNORD104 formed significantly larger tumors in nude mice compared to the control cells inoculated with the empty vector (Fig. 3G, *P < 0.05). Taken together, overexpression of SNORD104 significantly enhanced the proliferation, invasion, and migration of EC cells in vitro and promoted tumor growth in vivo.

### SNORD0104 upregulates and stabilizes PARP1 by promoting 2ʹ-O-methylation

**Fig. 4:**
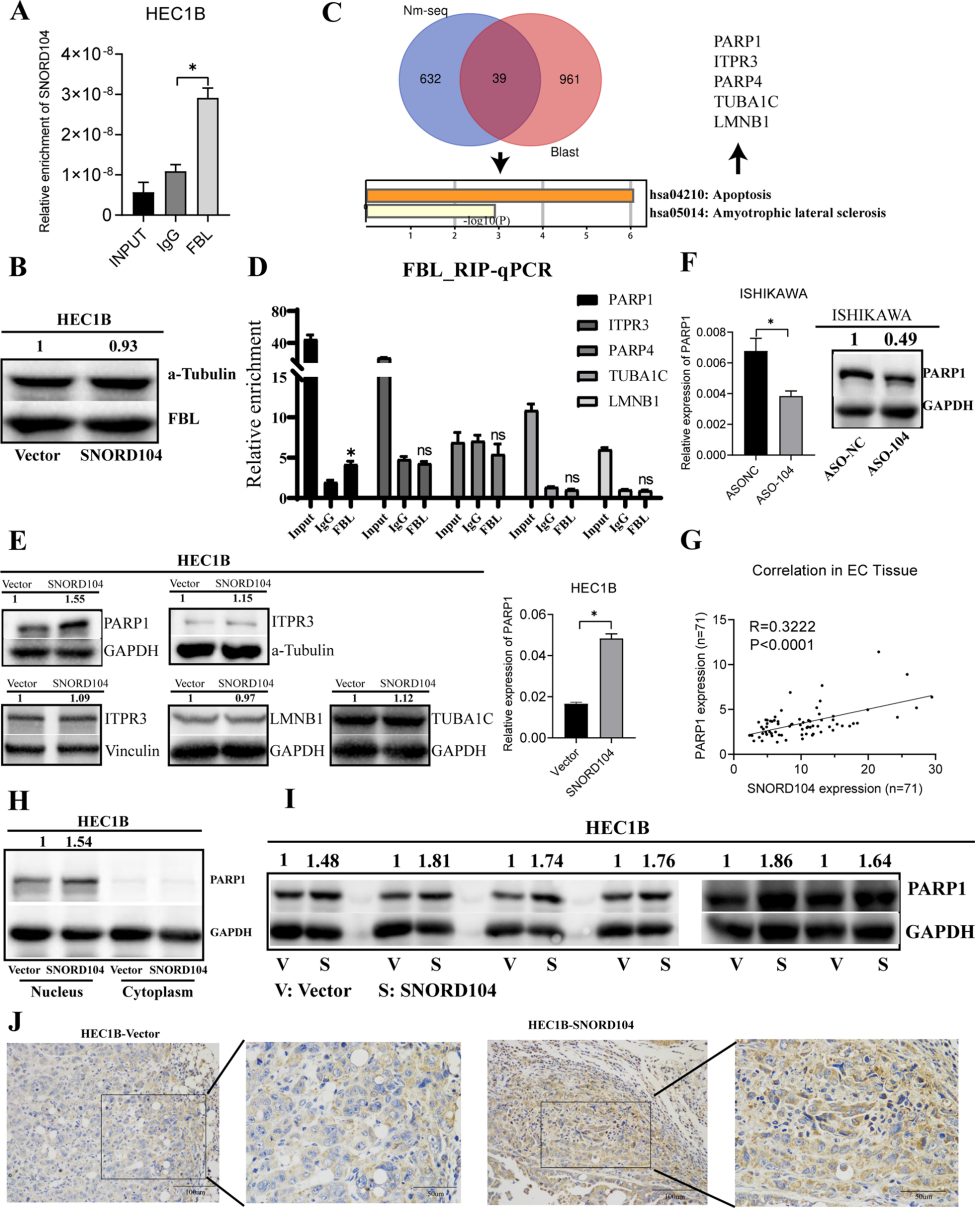
SNORD104 upregulated *PARP1* expression in vitro and in vivo. **A** Immunoprecipitation of SNORD104 using anti-FBL antibodies. **B** Immunoblot showing FBL protein levels in SNORD104-overexpressing HEC1B cells. **C** The 2ʹ-O-methylation level up-regulated RNA in the Nm-seq data set was intersected with the RNA data set of SNORD104-Blast, and the KEGG pathway enrichment analysis showed that the apoptotic pathway ranked first. **D** Anti-FBL antibody was used to perform RIP assay in HEC1B cells, and the mRNA enrichment of PARP1, ITPR3, TUBA1C, PARP4, LMNB1 was detected by qRT-PCR. **E** Evaluation of PARP1, ITPR3, TUBA1C, PARP4, LMNB1 protein levels after overexpression of SNORD104, and only PARP1 RNA and protein level were changed. **F** PARP1 mRNA and protein levels in SNORD104-knockdown Ishikawa cells. **G** SNORD104 and *PARP1* mRNA expression in EC tissues (n = 71) and the correlation between the expression levels (R = 0.3222, P < 0.0001). **H** PARP1 protein levels in the nuclear fraction of SNORD104-overexpressing cells. **I**, **J** PARP1 protein levels in the SNORD104-overexpressing subcutaneous xenografts in nude mice. *P < 0.05, ns means P > 0.05

The SNORDs and H/ACA box snoRNAs regulate gene expression via 2ʹ-O-methylation and pseudouridylation, respectively. Therefore, we hypothesized that SNORD104 might be involved in FBL-mediated 2ʹ-O-methylation. Indeed, the RIP assay of the lysates from SNORD104-overexpressing HEC1B cells using anti-FBL antibodies revealed significant enrichment of SNORD104 in the immuno-precipitate (Fig. 4A, *P < 0.05). However, SNORD104 overexpression did not affect the FBL protein level (Fig. 4B).

To identify potential target genes regulated by SNORD104-mediated 2ʹ-O-methylation, we performed NM-seq in the control and SNORD104-overexpressing cells. As shown in Fig. 4C, 2ʹ-O-methylation was upregulated (fold change ≥ 2) at 928 methylated sites (671 RNAs). It’s reported small nucleolar RNAs generally guide the modification of target RNAs through complementary base pairing. Therefore, we intersect the RNAs with up-regulated 2ʹ-O-methylation levels and the RNAs with complementary base pairing fragments of SNORD104. Next, we performed Kyoto Encyclopedia of Genes and Genomes (KEGG) pathway enrichment analysis of these hypermethylated RNAs. And the results showed that the apoptosis-related pathway ranked first (Fig. 4C). Further, we analyzed the enriched RNAs in apoptosis pathways, and the CPTAC database showed that their encoded proteins were upregulated in endometrial cancer tissues, including ITPR3, LMNB1, PARP1, PARP4, and TUBA1C (Additional file 3: Fig. S2A). Next, we used RIP assay to detect whether FBL protein binds to these 5 RNAs, and western blot to detect whether the proteins encoded by these 5 RNAs changed. The results show that only PARP1 mRNA binds to FBL protein (Fig. 4D, *P < 0.05), and only PARP1 mRNA and protein level changes (Fig. 4E, F, *P < 0.05). Therefore, we next analyzed the expression of *PARP1* in post-operative EC tissues and observed a positive correlation between SNORD104 and *PARP1* expression levels (Fig. 4G, *P < 0.05). PARP1 is primarily localized in the nucleus and is involved in DNA damage repair and ribosome biogenesis. Consistent with this, SNORD104 overexpression increased PARP1 protein levels in the nuclear fraction (Fig. 4H). In addition, the SNORD104-overexpressing xenografts also showed increased PARP1 protein levels (Fig. 4I, J).

**Fig. 5:**
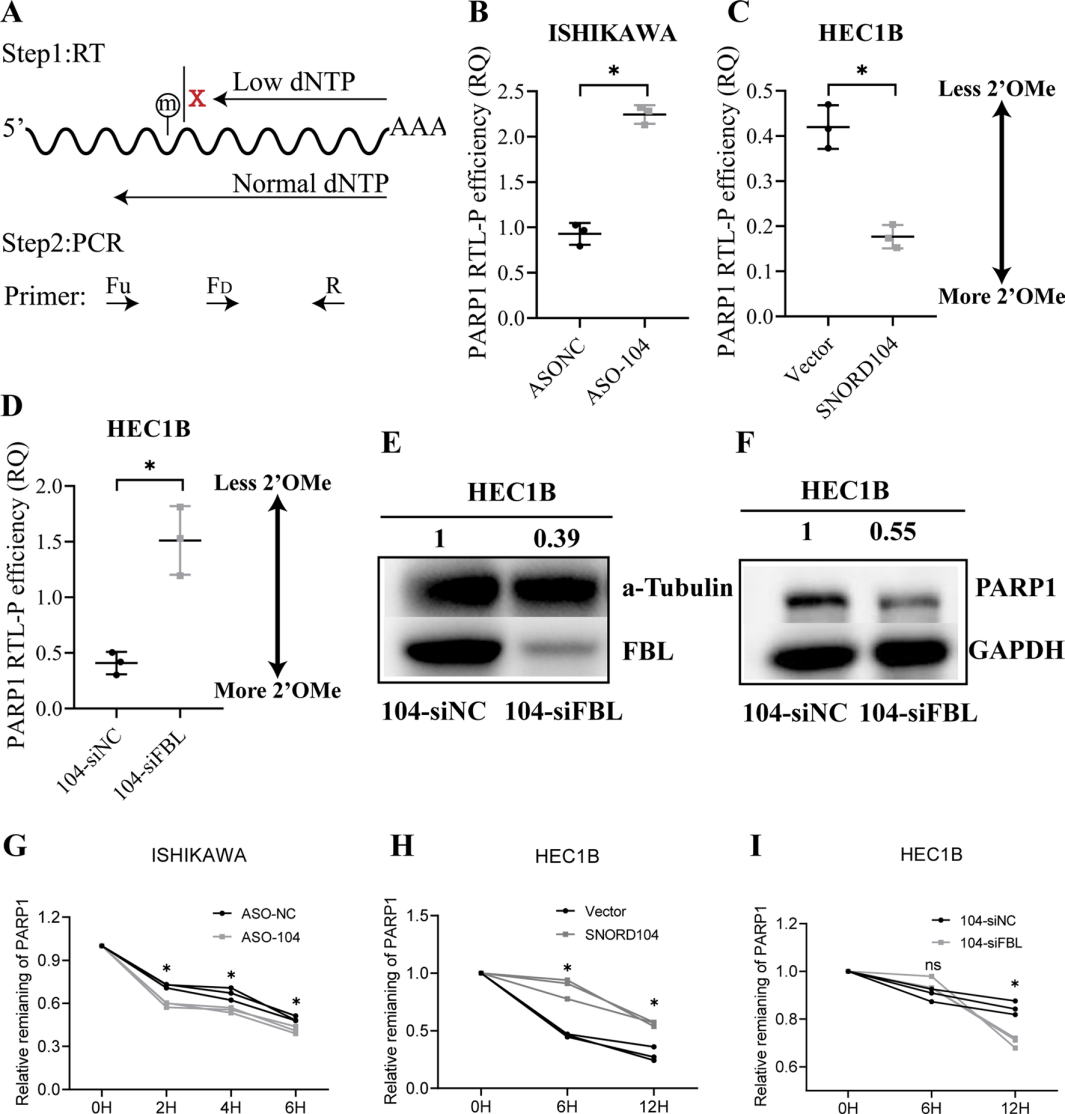
SNORD104 stabilizes *PARP1* mRNA via 2ʹ-O-methylation. (**A**) Schematic diagram of RTL-P experiment. **B** 2ʹ-O-methylated *PARP1* mRNA levels in SNORD104-knockdown cells. **C** 2ʹ-O-methylated *PARP1* mRNA levels in SNORD104-overexpressing HECB1 cells. **D** 2ʹ-O-methylated *PARP1* mRNA levels in SNORD104-overexpressing HECB1 cells with FBL knockdown. **E**, **F** PARP1 protein levels in SNORD104-overexpressing HECB1 cells with FBL knockdown. **G** *PARP1* mRNA stability in SNORD104-knockdown cells treated with Act D (5 μg/ml). **H** *PARP1* mRNA stability in SNORD104-overexpressing cells. **I** *PARP1* mRNA stability in FBL-knockdown cells. *P < 0.05

RTL-P assay was next performed to detect the level of 2ʹ-O-methylation in *PARP1* mRNA (Fig. 5A). SNORD104 knockdown and overexpression respectively decreased and increased the 2ʹ-O-methylation level of *PARP1* mRNA (Fig. 5B, C, *P < 0.05). Furthermore, *FBL* knockdown in the SNORD104-overexpressing HEC1B cells decreased both 2ʹ-O methylated *PARP1* mRNA as well as PARP1 protein levels (Fig. 5D, F, *P < 0.05). The biological role of ribose 2ʹ-O-methylation in mRNA is still unclear, although there are reports that it can improve the stability of nucleic acids against alkaline or enzymatic hydrolysis, most likely by altering their physical and chemical properties [17, 18]. To test this hypothesis, we treated the SNORD104-overexpressing or knockdown cells with transcription inhibitor actinomycin D. Compared to the control group, the SNORD104-overexpressing cells had significantly higher *PARP1* mRNA stability (Fig. 5G, *P < 0.05), while knocking down either SNORD104 or FBL reduced the stability of *PARP1* mRNA (Fig. 5H, I, *P < 0.05) (Additional file 3).

### SNORD104 promotes EC growth by regulating PARP1

**Fig. 6:**
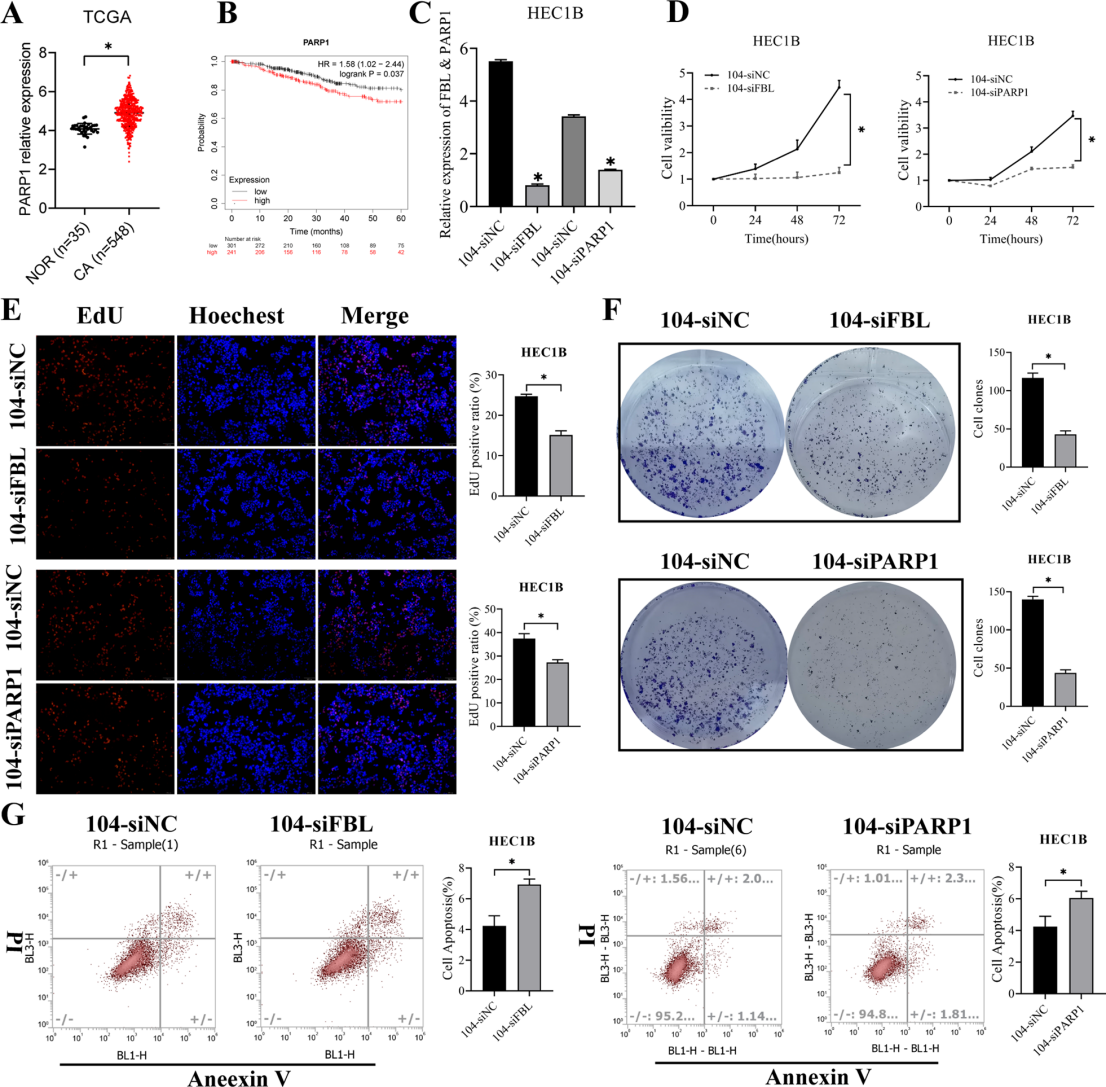
Knockdown of *FBL* or *PARP1* reversed the carcinogenic effect of SNORD104. **A** *PARP1* RNA levels in the EC tissues in the TGCA dataset. **B** Correlation between *PARP1* expression and 5-year overall survival (OS) of EC patients from the Kaplan–Meier Plotter (P = 0.037). **C** FBL and *PARP1* were knocked down in SNORD104-overexpressing HEC1B cells. The effect of *FBL* or *PARP1* knockdown on the **D**, **E** proliferation, **F** colony formation, and **G** apoptosis of SNORD104-overexpressing cells. *P < 0.05

*PARP1* functions as an oncogene in various cancers [19, 20]. A search of TCGA and CPTAC datasets [10] revealed that *PARP1* RNA and protein levels were significantly increased in EC tissues (Fig. 6A, Additional file 3: Fig. S2A, *P < 0.05). Furthermore, Kaplan–Meier analysis [11] showed the high expression of *PARP1* correlated with poor overall survival (Fig. 6B). To verify whether SNORD104 exerts its carcinogenic effect by regulating *PARP1*, knocked down *FBL* or *PARP1* in the HEC1B cells overexpressing SNORD104 (Fig. 6C, *P < 0.05). As expected, *FBL* or *PARP1*-silencing reversed the effects of SNORD104 on the proliferation (Fig. 6D, E, *P < 0.05), colony formation (Fig. 6F, *P < 0.05), and apoptosis of EC cells (Fig. 6G, *P < 0.05).

## Discussion

**Fig. 7:**
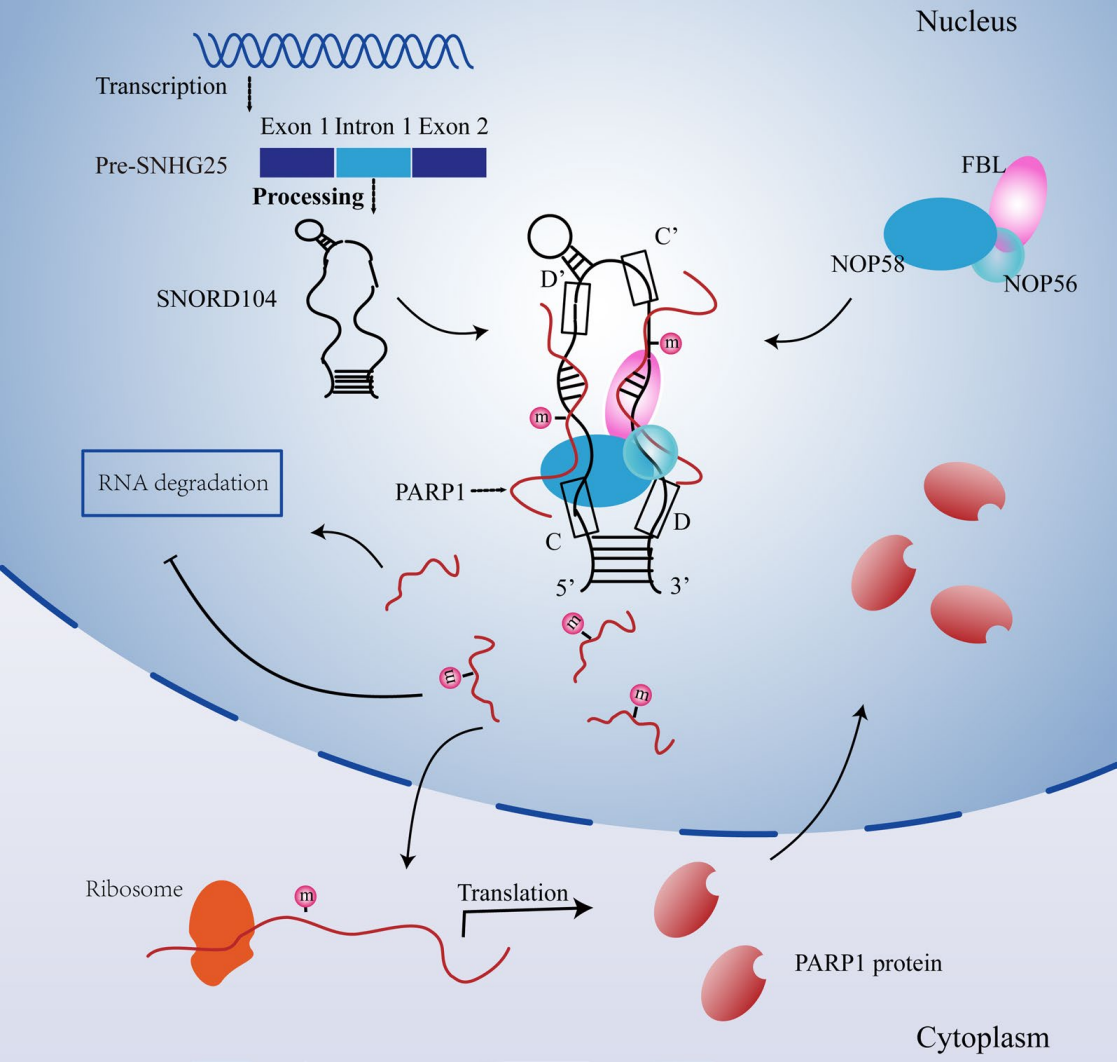
The mechanism of SNORD104 regulating *PARP1* expression through 2ʹ-O-methylation and promoting EC progression

Endometrial cancer (EC) occurrence and progression involve multiple genetic mutations and epigenetic changes, along with long-term stimulation of estrogen [21, 22]. In recent years, various non-coding RNAs, including long non-coding RNAs, circular RNAs, and small non-coding RNAs, such as microRNAs, piwi-interacting RNAs, and snoRNAs, have been identified that regulate tumor epigenetics. snoRNAs are dysregulated in multiple cancer and are associated with tumor cell growth, metastasis, and self-renewal [23]. For example, SNORD42A promotes acute myeloid leukemia cell proliferation by directing 18S rRNA 2'-O-methylation that promotes protein translation [24]. SNORD89 is highly expressed in ovarian cancer stem cells and promotes dryness of ovarian cancer cells by regulating the Notch1-c-Myc pathway [25]. SNORA74A activates PARP1-directed DExD-box helicase 21 (DDX21) ADP-ribosylation and increases the nuclear localization of DDX21 protein, thereby promoting ribosomal biogenesis and cancer cell growth [26]. However, little is known regarding the role of snoRNAs in EC. To identify the regulatory factors that play a role in the development of EC, we screened for the differentially expressed genes in EC tissues relative to the normal endometrial tissues using the Human Genome Map TCGA Database, and found that SNORD104 and its parent gene *SNHG25* were significantly upregulated in EC tissues. Although the expression levels of a few snoRNAs correlate with that of their host genes [27], the expression and biological functions of most snoRNAs seem to be independent of their host genes [28, 29]. In this study, we found that SNORD104, rather than its host gene *SNHG25*, promotes EC progression. SNORD104 increased the malignant potential of the EC cells in vitro and in vivo, and inhibited apoptosis, thus preliminarily confirming its pro-oncogenic effects in EC (Fig. 7).

The 2ʹ-O-methylation is a relatively conserved type of post-transcriptional modification that is abundant in rRNAs and tRNAs, and protects them against degradation [30]. High-throughput deep sequencing has revealed that 2ʹ-O-ribose methylation exists in the mRNAs and sncRNAs as well [31, 32]. In the present study, RIP experiments showed that SNORD104 binds to *FBL* protein without affecting the latter's expression levels, which further suggests that the biological function of SNORD104 may be exerted via the 2ʹ-O-ribose methylation catalyzed by FBL.

To further confirm this hypothesis, we performed NM-seq in HEC1B cells that overexpressed SNORD104 and identified 928 upregulated 2ʹ-O-methylation sites (671 RNAs). Since snoRNA mainly regulates the modification of target RNAs through complementary base pairing, we intersect the two data sets of Nm-seq and SNORD104-Blast. KEGG pathway enrichment analysis of these RNAs identified that the apoptosis pathway was significantly enriched. Then we analyzed the RNAs enriched in the apoptotic pathway through CPTAC database, and showed that the protein level *of ITPR3, LMNB1, PARP1, PARP4, and TUBA1C* were upregulated in endometrial cancer tissues. Next, RIP assays confirmed that FBL could bind to *PARP1* mRNA, but not the other 4 RNAs, and SNORD104 expression was positively correlated with *PARP1* expression in the EC tissues. Previous studies have shown that *PARP1* has two 2ʹ-O-methylation sites at CHR1: 226561928–226561929 and CHR1: 226549725–226549726 [33]. In contrast, our Nm-seq results showed that the modified site was located at CHR1: 226553726–22655372. Subsequently, we verified that SNORD104 regulates 2ʹ-O-methylation of *PARP1* mRNA in EC cells by RTL-P assay. In fact, SNORD104 overexpression or knockdown in EC cells significantly increased or decreased *PARP1* RNA and protein levels.

RNA methylation can regulate target genes [34] by influencing variable splicing [35], increasing RNA stability [36], and regulating RNA transport [37]. To determine the mechanism by which SNORD104-mediated 2ʹ-O-methylation upregulates PARP1 protein, we assessed *PARP1* mRNA stability after treatment with actinomycin D. SNORD104 overexpression or repression significantly enhanced or reduced *PARP1* mRNA stability, respectively. In addition, in HEC1B cells overexpressing SNORD104, *FBL* knockdown reduced the PARP1 protein levels. This suggested that SNORD104 formed a complex with FBL to target and regulate 2ʹ-O-methylation of *PARP1*, thereby increasing its protein level and enhancing its biological function. Finally, to verify that SNORD104 exerts its carcinogenic role by regulating the expression of *PARP1*, we knocked down *FBL* and *PARP1,* respectively, in SNORD104-overexpressing HEC1B cells, which expectedly reversed the oncogenic effects of SNORD104 in EC.

*PARP1*, the first member of the *PARP* gene family, is located on chromosome 1 1q42.12, and encodes the chromatin-related enzyme, poly (ADP ribosyl) transferase. PARP1 participates in the poly (ADP-ribosylation) modification induced by DNA damage [38–40]. In addition, PARP1 is an important factor in nucleolar biogenesis. Under non-stress conditions, about 40% of PARP1 molecules are located in the nucleolus [41], where it mediates ribosomal biosynthesis by controlling pre-rRNA processing, post-transcriptional modification, and assembly of pre-ribosomal subunits [42]. Recent studies reported that PARP1 plays a very extensive role in regulating the occurrence and development of tumors, not only participating in cancer cell proliferation, but also participating in metastasis. For instance, it catalyzes poly ADP-ribosylation of *STAT3* (encoding signal transducer and activator of transcription 3) in various cancer cells and subsequently promotes *STAT3* dephosphorylation, resulting in decreased transcription of STAT3 and its target gene *PDL1* (encoding programmed cell death 1 ligand 1) [43]. In breast cancer cells, PARP1 competes with histone H1 to maintain transcription of the oncogene *CCND1* (encoding cyclin D1), and also acts as a transcriptional coactivator of GATA binding protein 3 (GATA3), thus promoting the transcription of *CCND1* [44]. In prostate cancer and kidney cancer, PARP1 is closely related to the metastasis of tumor cells [45, 46]. Thus, PARP1 participates in the occurrence and development of cancer through complex regulatory mechanisms.

## Conclusions

SNORD104 is upregulated in EC and promotes tumor growth by inducing 2ʹ-O-methylation of *PARP*1, which enhances the latter’s expression and biological function. Our findings provide new insights into the mechanisms underlying the development and progression of EC and identify novel therapeutic targets for individualized treatment.

## Supplementary Information

**Additional file 1: Table S1.** Correlation of SNORD104 expression with different clinicopathological features of endometrial cancer.

**Additional file 2: Fig. S1.** Characteristics of *SNHG25* in endometrial cancer. *SNHG25* expression in (A) EC tissues and (B) cell lines. (C&D) *SNHG25* was knocked down in Ishikawa cells and overexpressed in HEC1B cells. (E, F) Effect of *SNHG25* knockdown on the viability and apoptosis of Ishikawa cells. (G, H) Effect of* SNHG25 *overexpression on the viability and apoptosis of HEC1B cells. *P < 0.05, ns means P>0.05.

**Additional file 3: Fig. S2.** SNORD104 potential target RNAs in endometrial cancer. (A) Protein expression of ITPR3, LMNB1, PARP1, PARP4, TUBA1C in endometrial cancer (n=100) and normal endometrial tissue (n=31).

## Data Availability

The datasets supporting the conclusions of this article are included within the article and its additional file. For any further of the data requests, please contact the corresponding author.
