# Supplementary material for: C/D box small nucleolar RNA SNORD104 promotes endometrial cancer by regulating the 2ʹ-O-methylation of PARP1
Source: J Transl Med. 2022 Dec 24;20:618. doi: 10.1186/s12967-022-03802-z (PMC9790134; doi:10.1186/s12967-022-03802-z)
Supplement: Supplementary file 1 — Additional file 1: Table S1. Correlation of SNORD104 expression with different clinicopathological features of endometrial cancer. [file 12967_2022_3802_MOESM1_ESM.doc]

**Table S1:** Correlation of **SNORD104** expression with different clinicopathological features of endometrial cancer

| **Clinicopathological features** | **N** | **SNORD104 expression / U6** | ***P* value** |
| --- | --- | --- | --- |
|
| **FIGO stages** |  |  | ***5.445E-07*** |
| I- II | 46 | 4.45±2.65 |  |
| III-IV | 13 | 9.71±4.29 |  |
| **Pathology classification** |  |  | ***0.009*** |
| Well+Mod | 44 | 4.65±2.86 |  |
| Poor | 15 | 7.61±5.19 |  |
| **Age** |  |  | 0.67 |
| ≤ 53 | 29 | 5.18±3.13 |  |
| > 52 | 30 | 5.80±4.38 |  |
| **Lymph node metastasis** |  |  | ***2.222E-07*** |
| No | 50 | 4.40±2.78 |  |
| Yes | 9 | 10.94±4.08 |  |
| **Infiltration** |  |  | ***0.009*** |
| Shallow | 33 | 4.28±2.79 |  |
| Deep | 26 | 6.88±4.57 |  |
| Vascular invasion |  |  | 0.056 |
| No | 29 | 4.43±2.87 |  |
| Yes | 30 | 5.72±4.36 |  |
| Bold and Italics means P < 0.05. |  |  |  |
|  | | | |
