# Supplementary figures and images for: C/D box small nucleolar RNA SNORD104 promotes endometrial cancer by regulating the 2ʹ-O-methylation of PARP1
Source: J Transl Med. 2022 Dec 24;20:618. doi: 10.1186/s12967-022-03802-z (PMC9790134; doi:10.1186/s12967-022-03802-z)

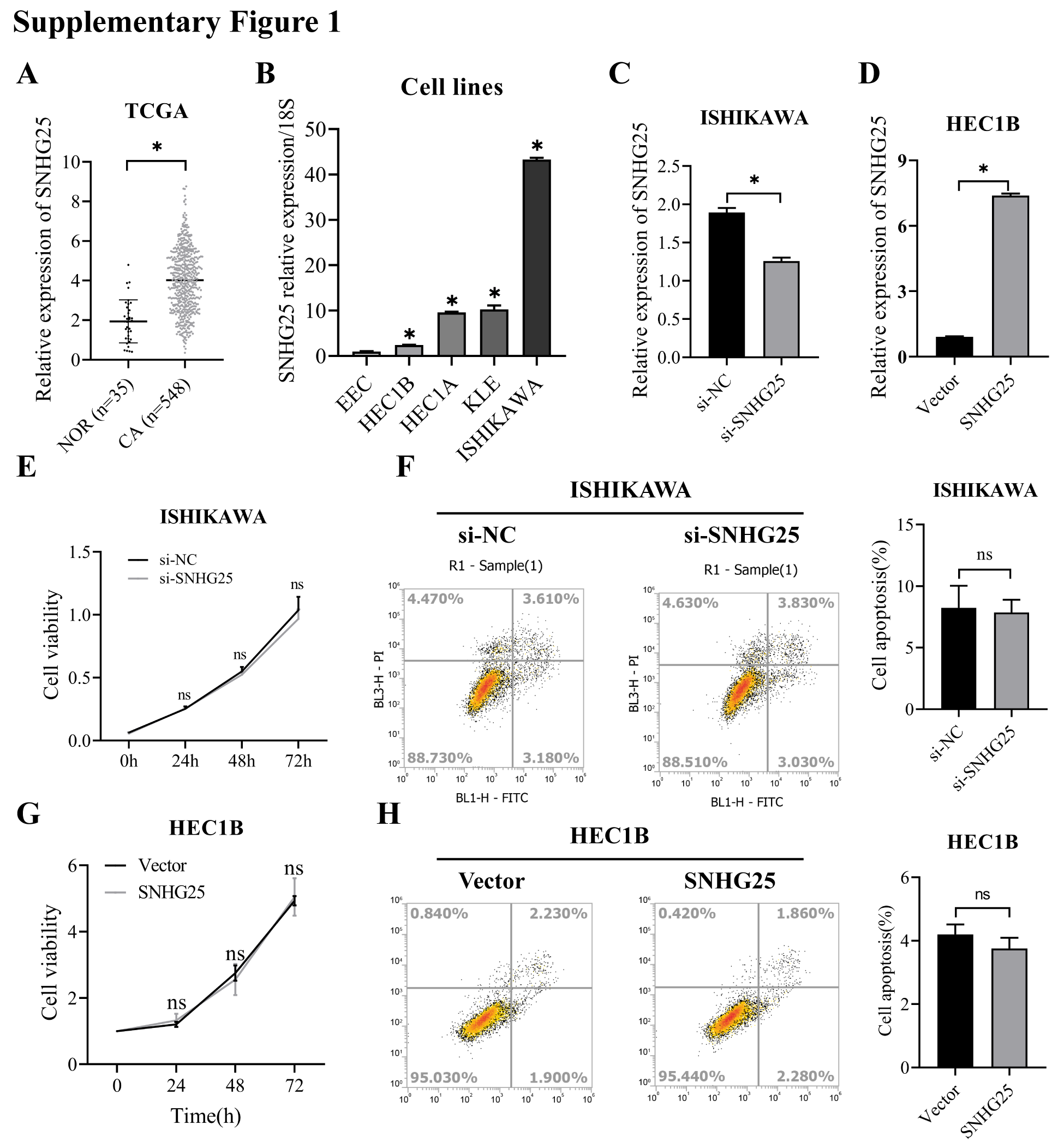

Supplement: Supplementary file 2 — Additional file 2: Fig. S1. Characteristics of SNHG25 in endometrial cancer. SNHG25 expression in (A) EC tissues and (B) cell lines. (C&D) SNHG25 was knocked down in Ishikawa cells and overexpressed in HEC1B cells. (E, F) Effect of SNHG25 knockdown on the viability and apoptosis of Ishikawa cells. (G, H) Effect of SNHG25 overexpression on the viability and apoptosis of HEC1B cells. *P < 0.05, ns means P>0.05. [file 12967_2022_3802_MOESM2_ESM.tif]

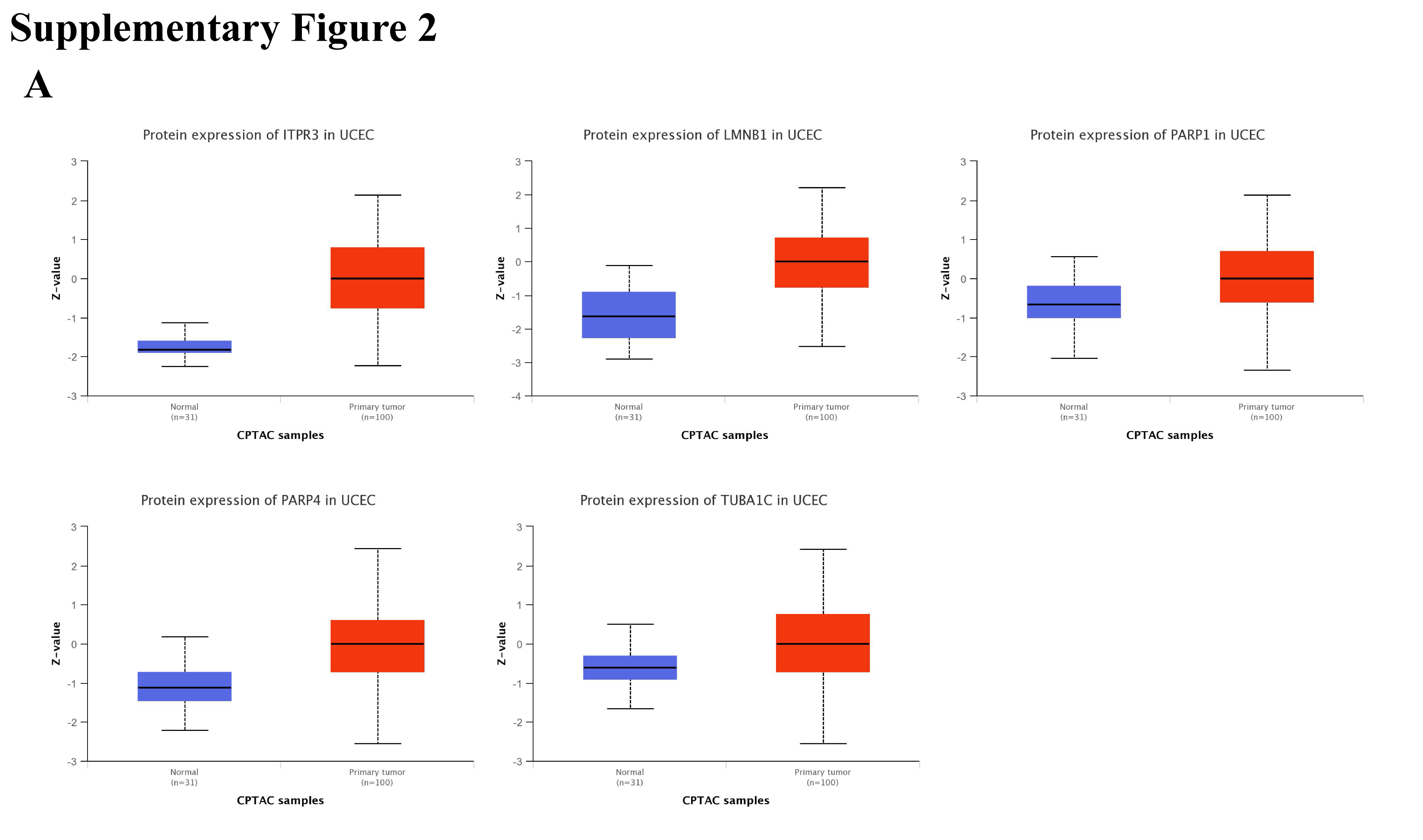

Supplement: Supplementary file 3 — Additional file 3: Fig. S2. SNORD104 potential target RNAs in endometrial cancer. (A) Protein expression of ITPR3, LMNB1, PARP1, PARP4, TUBA1C in endometrial cancer (n=100) and normal endometrial tissue (n=31). [file 12967_2022_3802_MOESM3_ESM.tif]
